# Evolution and biogeography of the endemic *Roucela* complex (Campanulaceae: Campanula) in the Eastern Mediterranean

**DOI:** 10.1002/ece3.1791

**Published:** 2015-10-28

**Authors:** Andrew A. Crowl, Clayton J. Visger, Guilhem Mansion, Ralf Hand, Hsin‐Hui Wu, Georgia Kamari, Dimitrios Phitos, Nico Cellinese

**Affiliations:** ^1^ Florida Museum of Natural History University of Florida Gainesville Florida; ^2^ Department of Biology University of Florida Gainesville Florida; ^3^ Botanischer Garten und Botanisches Museum Berlin‐Dahlem Freie Universität Berlin Berlin Germany; ^4^ Department of Biology University of Patras Patras Greece

**Keywords:** Aegean Archipelago, Campanulaceae, continental islands, *drabifolia* complex, endemism, Mediterranean, *Roucela* clade

## Abstract

At the intersection of geological activity, climatic fluctuations, and human pressure, the Mediterranean Basin – a hotspot of biodiversity – provides an ideal setting for studying endemism, evolution, and biogeography. Here, we focus on the *Roucela* complex (*Campanula* subgenus *Roucela*), a group of 13 bellflower species found primarily in the eastern Mediterranean Basin. Plastid and low‐copy nuclear markers were employed to reconstruct evolutionary relationships and estimate divergence times within the *Roucela* complex using both concatenation and species tree analyses. Niche modeling, ancestral range estimation, and diversification analyses were conducted to provide further insights into patterns of endemism and diversification through time. Diversification of the *Roucela* clade appears to have been primarily the result of vicariance driven by the breakup of an ancient landmass. We found geologic events such as the formation of the mid‐Aegean trench and the Messinian Salinity Crisis to be historically important in the evolutionary history of this group. Contrary to numerous past studies, the onset of the Mediterranean climate has not promoted diversification in the *Roucela* complex and, in fact, may be negatively affecting these species. This study highlights the diversity and complexity of historical processes driving plant evolution in the Mediterranean Basin.

## Introduction

Spatial patterns of biological diversity are shaped by numerous factors, including biotic interactions, habitat heterogeneity, area, climatic constraints, isolation, and anthropogenic events (Huston 1994). Uncovering the relative contributions of these factors and evolutionary dynamics responsible for driving endemism is essential to understanding plant diversity and may have important implications for conservation.

Endemic species are nonrandomly distributed across terrestrial habitats and appear to be concentrated in specific regions, or “hotspots” of biodiversity (de Candolle 1875; Kruckeberg and Rabinowitz 1985; Myers et al. 2000), such as the Mediterranean Basin (e.g., Médail and Quézel 1997; Thompson 2005). The complex, but well understood, climatic and geologic history of this region provides an ideal setting for studying endemism, evolution, and biogeography.

While the western Mediterranean Basin has been relatively well studied (e.g., Mansion et al. 2008, 2009), the eastern basin remains poorly understood. With a high degree of endemism and both oceanic and continental islands present, this region affords a unique opportunity to better understand the processes leading to endemism on these distinctly different classes of islands within the same geographic area.

### Oceanic and continental islands

Historically, islands were viewed as fragments of continents until Charles Darwin and Alfred Russell Wallace made a distinction between continental islands, which have had a past connection with the mainland, and oceanic islands – those that have arisen from the ocean and have no history of continental connection (Darwin 1859; Wallace 1902).

These two types of islands are fundamentally different, both geologically and biologically. Oceanic islands are formed by volcanic activity or tectonic events and arise from the ocean, never having been in contact with an organismal source. They therefore have initially empty ecological niche space. Continental islands, in contrast, are formed by tectonic events or rising sea levels causing the breakup or isolation of a fragment from the continent and contain a balanced flora and fauna at the time of isolation. Crete, Kasos, Karpathos, Rhodes, and the numerous small islands off the west coast of Turkey represent continental systems included in this study while Cyprus is of oceanic origin.

### Geologic and climatic history of the eastern Mediterranean Basin

The geologic and climatic history of the eastern Mediterranean Basin since the Miocene is a complex combination of tectonic events, sea‐level changes, volcanism, and a trend toward summer drought and increased seasonality. All of these events have had a profound effect on the flora and fauna of the area (Thompson 2005). Below we lay out those that had the largest impact on biogeographic patterns in the eastern Mediterranean and are, thus, potential drivers of diversification and current distribution patterns in this region.

A continuous landmass (termed Ägäis) stretched from present day Turkey to present day Greece, until approximately 12 Ma, when rising sea levels and tectonic activity caused it to break up (Creutzburg 1963; Dermitzakis 1990; Triantis and Mylonas 2009). This began the formation of the Aegean Archipelago and formed many of the continental islands in the eastern Mediterranean. During this time (12–9 Ma), the MAT (mid‐Aegean trench) formed, causing a tectonic split between Crete and Karpathos, stretching northward (Creutzburg 1963; Dermitzakis 1990).

With the closure of the Mediterranean's connection with the Atlantic Ocean approximately 5.96 Ma, a major desiccation of the Mediterranean Basin occurred (Hsu et al. 1973; Krijgsman et al. 1999). The MSC (Messinian Salinity Crisis) led to the reconnection of many islands to each other and to the mainland, potentially facilitating dispersal between previously isolated areas. Approximately 5.33 Ma, this barrier was broken and a rapid reflooding of the basin occurred, leaving many islands once again isolated (Krijgsman et al. 1999). This event led to extreme aridity and likely caused significant extinction in subtropical lineages and diversification within arid‐adapted groups (Fiz‐Palacios et al. 2010; Jimenez‐Moreno et al. 2010).

Cyprus has an incredibly complex paleogeographic history and is one of the most isolated islands in this region (Moores et al. 1984). The current configuration of the island is, in fact, the result of a connection between two separate oceanic islands – the Troodos Massif to the southwest and the Kyrenia Range to the north. The Troodos Massif was likely an island by the Late Miocene, at which time the Kyrenia Range began to rise (Hadjisterkotis et al. 2000). The collision of these two mountain ranges was followed by the uplift of the central and coastal areas, resulting in the present island formation during the Pliocene–Pleistocene transition (Hadjisterkotis et al. 2000; Yerkes 2012). The Troodos Massif is dominated by diabase and serpentine soil, while the Kyrenia Range is composed primarily of limestone.

More recent, and perhaps less dramatic, are the eustatic sea‐level changes during the Pleistocene. Glacial and interglacial periods saw many islands in close proximity to each other and the mainland going through periods of isolation and reconnection. For example, land bridges likely connected many of the eastern Aegean islands with each other and with mainland Turkey during the last glacial maximum, when the sea level was approximately 120 m below present levels (Shackleton 1987).

Climatic fluctuations have been quite dramatic and significant in this area. Subtropical conditions persisted through the early Miocene (23–16 Ma) with high summer rainfall and little seasonal temperature changes. A gradual decrease in summer rainfall and a trend toward increased aridification and seasonality began in the middle Miocene (9–8 Ma) and continued into the Pliocene, leading to the establishment of the current Mediterranean climate (3.4–2.8 Ma; Suc, 1984).

### *Roucela* complex

In this study, we focus on the *Roucela* complex – referred to as the *drabifolia* species complex by Carlström (1986) – which includes small, herbaceous, annual *Campanula* species restricted to the Mediterranean Basin, characterized by the presence of unappendaged calyx lobes (Carlström 1986; Lammers 2007). In the last available revision, Carlström (1986) disentangled the group, formally recognizing 12 morphological species. One additional species, *Campanula lycica*, was later described and added to this complex by Tan and Sorger (1986).

This taxonomically difficult group has historically been considered at various ranks, including its own genus distinct from *Campanula* (*Roucela*; Du Mortier 1822), and a subgenus of *Campanula* (Damboldt 1976; Lammers 2007). Here we repurpose the old genus name, *Roucela*, in reference to the clade that includes the taxa subject of our study.

**Figure 1:**
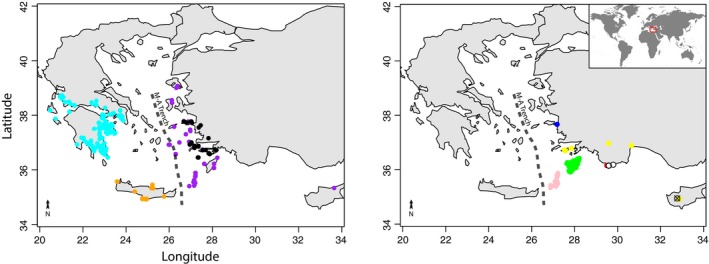
Occurrence map. Occurrence maps for taxa in the *Roucela* complex based on field observations and herbarium collections. Light blue: *Campanula drabifolia*. Orange: *Campanula creutzburgii*. Purple: *Campanula delicatula*. Black: *Campanula simulans*. Green: *Campanula rhodensis*. Pink: *Campanula pinatzii*. Dark blue: *Campanula raveyi*. Yellow: *Campanula podocarpa*. Red: *Campanula kastellorizana*. White: *C. lycica*. “X”: *Campanula veneris*. Occurrences of the widespread *Campanula erinus* not shown. Dashed line indicates approximate location of the mid‐Aegean trench.

The current distribution and high level of endemism in the *Roucela* complex makes it an ideal model for understanding historical drivers of speciation and endemism in the Mediterranean Basin. Species in the group are primarily found in the eastern Mediterranean, with very restricted distributions, many endemic to a single or a few islands in the Aegean Archipelago, western Turkey, or Cyprus (Fig. 1). An exception is the widespread *Campanula erinus*, found from the Azores, southern Europe and northern Africa, to the Arabian Peninsula – an area broadly corresponding to the Mediterranean climate zone. Interestingly, this is the only known self‐compatible taxon.

### Summary

Here we present a phylogeny of the *Roucela* complex, inclusive of all 13 taxa traditionally recognized in this group. We assess the monophyly of the complex and infer potential gene flow between species by utilizing five plastid and two nuclear loci recently found to be informative in the Campanuloideae (Crowl et al. 2014). We use molecular dating, biogeographic reconstruction, and diversification analyses to establish timing of major splitting events and generate hypotheses regarding potential drivers (and inhibitors) of diversification in this clade. Finally, we infer potential climatic niche space of *Roucela* species by employing ecological niche modeling techniques to test the hypothesis that climatic constraints are responsible for the narrow species distributions observed.

## Materials and Methods

### Taxon sampling and DNA amplification

Taxon sampling for this study included multiple accessions of all 13 representatives of the *Roucela* complex (Fig. S1 in Supporting Information) as defined by Carlström (1986) and Tan and Sorger (1986). In order to estimate divergence times, infer the placement of these taxa with other *Campanula* species, and test the monophyly of the group, we analyzed *Roucela* accessions within the context of a larger Campanuloideae dataset. Sampling for these analyses followed Crowl et al. (2014).

Total genomic DNA was extracted from field‐collected, silica‐dried leaf material and herbarium specimens following a modified CTAB (cetyltrimethyl ammonium bromide) extraction protocol (Doyle and Doyle 1987). We amplified and sequenced five plastid regions (*atpB*,* matK*,* petD*,* rbcL*, and the *atpB*–*rbcL* intergenic spacer region) and two low‐copy nuclear loci (*PPR11* and *PPR70*) from the pentatricopeptide repeat (PPR) gene family.

All sequences were amplified following Crowl et al. (2014). Because orthology of nuclear loci has been assessed by this previous study, PCR products of appropriate size (approximately 800–1000 bp) were sequenced directly. All sequences have been deposited in GenBank (Fig. S1).

### Phylogenetic analysis

**Figure 2:**
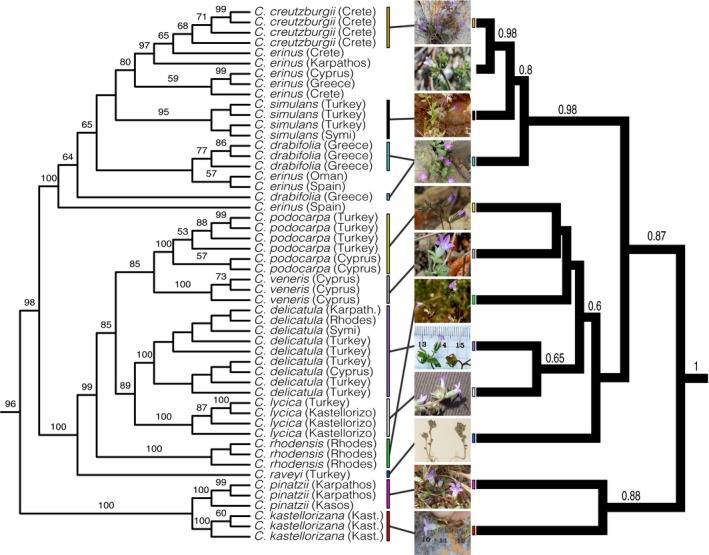
Concatenated and species trees. Maximum‐likelihood tree (left) and species tree from *BEAST analysis (right) for the *Roucela* clade. Bootstrap support (>50%) and posterior probability values (>0.50) given above branches. Photograph of *Campanula podocarpa* by Charalambos Christodoulou. Remaining photographs by AA Crowl.

We used jModelTest 2 (Darriba et al. 2012) to determine appropriate models of molecular evolution for individual loci. Maximum‐likelihood analyses were run in RAxML (version 7.0.4; Stamatakis 2006) with 1000 bootstrap replicates. Given that individual gene trees provided congruent results, we combined the five plastid and the two nuclear markers into independent datasets in order to compare histories from these different genomes. We then constructed a combined (concatenated) dataset including all seven markers. The combined dataset included only accessions for which more than one marker was available (Fig. 2). *Cyphia elata* was used as the out‐group for plastid and combined analyses while *Solenopsis minuta* served as the out‐group for nuclear analyses due to availability of sequences. Datasets were partitioned by gene. The combined alignment is available through the Dryad repository (doi: 10.5061/dryad.v6p3h).

Because phylogenetic analysis placed *Campanula scutellata* outside the *Roucela* clade (see Results), this taxon was excluded from all subsequent analyses.

### Species tree

Multispecies coalescent approaches are likely to give more accurate results for multiple unlinked partitions when compared to analyses of concatenated datasets (Maddison and Knowles 2006). We conducted species tree analyses using a Bayesian multispecies coalescent approach implemented in *BEAST (BEAST v.1.8.0; Heled and Drummond 2010). Our dataset consisted of three independent loci: one plastid (all plastid markers were treated as a single locus) and two nuclear loci. Simulation studies (Maddison and Knowles 2006; McCormack et al. 2009; Heled and Drummond 2010) have found that three independent loci across multiple individuals will accurately recover the species tree for groups with divergence times much younger than we expect in this study. We used a Yule prior for the species tree and applied the best‐fit model identified by jModelTest 2 (Darriba et al. 2012) for each locus. This analysis was run with a chain length of 10^7^, and a 10% cut‐off was used for the burn‐in.

### Molecular dating

Molecular dating analyses were performed under a relaxed molecular clock to estimate divergence times for the *Roucela* complex. In order to utilize a fossil constraint, we included these taxa within the context of a larger Campanuloideae dataset.

**Figure 3:**
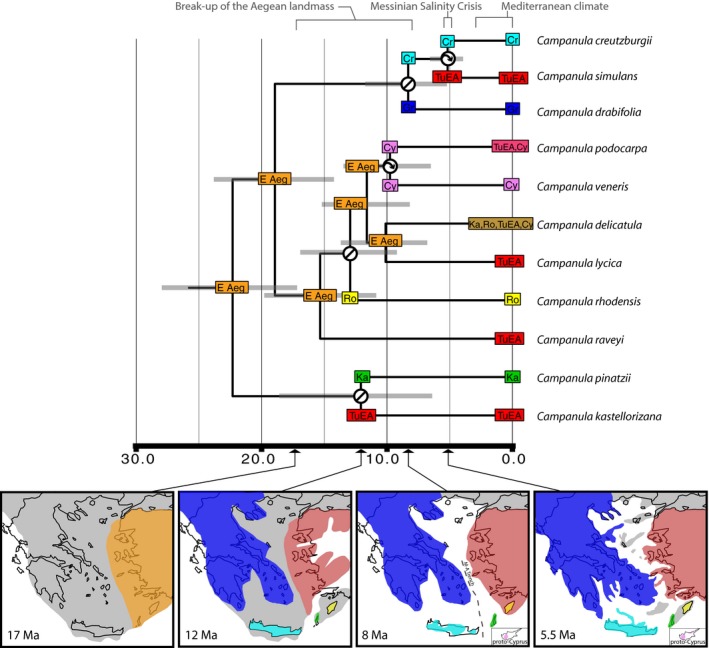
Chronogram of the *Roucela* clade with ancestral range estimation. Summary chronogram from Bayesian dating analysis (BEAST). Out‐groups not shown. Monophyletic species have been reduced to a single lineage; *Campanula erinus* excluded. Current distribution of each taxon is indicated on the terminals of the tree. Cr, Crete; TuEA, Turkey and East Aegean islands; Gr, mainland Greece; Cy, Cyprus; Ro, Rodos; Ka, Karpathos and Kasos; E Aeg, east Aegean landmass. Internal colored squares indicate most likely ancestral area recovered by BioGeoBEARS under the DEC+J model. Corners represent ranges immediately following a speciation event. Circles with an arrow denote dispersal events, while circles with a line denote vicariance. Horizontal gray bars represent 95% HPD confidence intervals. Maps are paleogeographic reconstructions of the Aegean area through time redrawn from Kasapidis et al. (2005) and Parmakelis et al. (2006) with water depicted as white and land shaded. Areas are colored as coded in the biogeographic analysis, showing connections and isolation through time. See Figure S3 for full tree including all accessions.

BEAST (v.1.8.0; Heled and Drummond 2010) analyses were run under an uncorrelated lognormal model – which assumes no autocorrelation of rates – for 10^8^ generations, logging parameters every 1000 generations, and assuming a Yule process. We allowed BEAST to simultaneously infer tree topology and divergence dates with no constraints on topology except for the out‐group. We used our three independent loci (plastid, PPR11, PPR70) as input; jModelTest 2 (Darriba et al. 2012) was used to find the best‐fit model for each locus (GTR + Γ + I for all partitions). Tracer v.5.0 (Drummond et al. 2012) was used to assess ESS (effective sample sizes values) for estimated parameters and to assess burn‐in. Ten percent of trees were removed as burn‐in, and summary statistics were calculated from the remaining trees using TreeAnnotator v.1.7.4 (Drummond et al. 2012) to provide a summary tree (Fig. 3).

The fossil record of the Campanulaceae is especially scarce (Lammers 2007). However, reliable fossil seeds do exist for *Campanula*. These fossils are identified as *Campanula* sp. and *Campanula paleopyramidalis* from the Miocene (17–16 Ma) of the Nowy Sacz Basin in Poland (Lancucka‐Srodoniowa 1977, 1979). See Crowl et al. (2014) for further discussion.

A lognormal prior distribution was applied to the most recent common ancestor of *Campanula pyramidalis* and *Campanula carpatica* with a mean of 5.0, stdev of 1.0, and an offset of 16, giving a minimum age constraint for the fossil node. Furthermore, two additional calibration points were used to constrain deeper nodes. We used previously obtained dates from a recent study (Bell et al. 2010), which used numerous fossil calibrations to date major clades within angiosperms. Date ranges from the 95% highest posterior densities were used to constrain the node representing the split between the Campanuloideae and the rest of the family (41–67 Ma) as well as the crown of the Campanuloideae (28–56 Ma). Normal distribution priors were placed on both of these nodes, using the mean from each range reported in Bell et al. (2010) and a stdev of 5.0.

Additionally, we estimated divergence times using a multispecies coalescent (species tree) approach in *BEAST (Fig. S5). Although species tree methods are often preferable to concatenation approaches, they may not be appropriate for estimating divergence times if gene flow is present between species, as is likely in the *Roucela* complex (see Results). Estimation errors in divergence times can be greatly impacted by the migration of even a single individual (Leaché et al. 2014). We confirmed this assertion (Fig. S5) and found divergence dates an order of magnitude younger than the BEAST analysis and previous estimates (Mansion et al. 2012; Crowl et al. 2014).

### Biogeographic analysis

We estimated ancestral ranges using the BioGeoBEARS package (Matzke 2013) in R. All models, including DEC, BAYESAREALIKE, and DIVALIKE, were tested. Additionally, this program implements a founder‐event speciation parameter (+J), which may be important in island systems (Matzke 2013). We used likelihood‐ratio tests and AIC (Akaike information criterion) values to compare the fit of these models to the data. Each taxon was coded for presence/absence in six geographic areas: mainland Greece, Crete, Kasos/Karpathos, Rhodes, Cyprus, and Turkey. Turkey was coded to include the islands immediately off the west coast, which have been connected to the mainland in recent geologic history relative to the diversification of the *Rouclea* taxa. For this analysis, we used the BEAST maximum clade credibility tree with out‐groups removed and species collapsed to a single representative lineage. The maximum number of ancestral areas to be reconstructed at each node was set to six.

We conducted three analyses in BioGeoBEARS: (1) a non–time‐stratified analysis allowing unconstrained dispersal between all areas through time; (2) a time‐stratified analysis, with variable dispersal rates across five time intervals by subdividing the phylogeny at 0.5, 5.33, 5.96, 12, and 25 Ma, corresponding to major changes in the connections and formation of landmasses in the eastern Mediterranean Basin; and (3) due to the uncertainty in the placement and monophyly of *C. erinus*, we omitted this taxon from the chronogram and conducted a stratified analysis identical to analysis (2), above.

### Ecological niche modeling

We used ecological niche modeling techniques to estimate potential climatic niches for six species within the *Roucela* clade in order to better understand the nature of endemism in the group and test the hypothesis that climatic constraints may be responsible for the narrow distributions observed. Occurrence data were gathered from both field observations and museum collections aggregated in GBIF (http://www.gbif.org). Duplicate localities and points that were collected well outside of the expected range of a taxon (likely misidentified specimens) were removed. Climatic datasets used had a resolution of 1 km^2^. We therefore restricted occurrence points to a single locality per 1 km^2^ in order to avoid spatial autocorrelation and sampling bias using ENMTools (Warren et al. 2010). Number of individuals varied from 21 (*Campanula creutzburgii*) to 124 (*Campanula drabifolia*) after the above quality control. Due to insufficient sampling (fewer than 10 occurrences), we were unable to confidently include the rare, narrow endemics *Campanula kastellorizana*,* C. lycica*,* Campanula podocarpa*,* Campanula raveyi,* and *Campanula veneris*. *Campanula erinus* was excluded due to its widespread distribution, occurring beyond the eastern Mediterranean (and, thus, beyond the scope of this study) and insufficient sampling across its range.

Twenty global climate and elevation layers with a spatial resolution of 1 km^2^ were obtained from the WorldClim database (Hijmans et al. 2005). Layers were clipped to the eastern Mediterranean Basin using QGIS (Open Source Geospatial Foundation Project, 2014), and 10,000 randomly distributed points were sampled across each layer. The 10,000 values from the 20 layers were tested for correlation using JMP Pro v.11. (SAS Institute Inc, 1989–2007) Climate layers found to be highly correlated (>0.70 Pearson's correlation coefficient) were excluded from subsequent analyses. When two layers were found to be highly correlated, one was removed. This approach was implemented for all pairwise comparisons until all remaining layers were below the 0.70 correlation threshold.

We used Maxent (v.3.3.3k; Phillips et al. 2006) to infer potential climatic niches. This method requires occurrence‐only data and has been found to perform well with sample sizes as low as 10, a useful feature when studying narrow endemics (Hernandez et al. 2006; Pearson et al. 2007). Default settings were used, with the following exceptions: 10 subsampled replicates, test percentage of 15%, and 5000 maximum iterations. Statistical evaluation of niche and distribution model predictions was performed using the AUC (area under the curve) of the receiver operating characteristic statistic, which provides a way to assess the ability of the model to correctly predict distributions of the training points.

### Diversification

We utilized a number of diversification methods implemented in R (v.3.1.0; R Core Team 2013) to better understand patterns of the timing and tempo of lineage diversification within the *Roucela* clade and test the hypothesis that allopatric speciation caused by the breakup of the Aegean landmass is responsible for much of the diversification within the group. The chronogram from the BEAST analysis was trimmed to include only a single accession for all *Roucela* taxa. *Campanula erinus* was also reduced to a single accession, giving a topology that approximated the species tree. Although this currently recognized species may represent multiple cryptic taxa, until further studies can disentangle this complex with confidence, we chose to follow current taxonomy and represent *C. erinus* as a single lineage for these analyses. LTT (Lineage‐through‐time) plots were constructed for the post‐burn‐in posterior distribution of trees using the APE package (v.3.1‐1; Paradis et al. 2004). We calculated the gamma statistic (Pybus and Harvey 2000) as implemented in GEIGER (Harmon et al. 2008) to test whether diversification rates have been constant through time for this clade.

We then further explored diversification rates through time following the approach of Simpson et al. (2011). This method uses the LASER package (Rabosky 2006) and calculates diversification rates across the tree by estimating the number of nodes and their corresponding branch lengths within a sliding window. We used a window width of five million years.

A variety of diversification models were then fit to our data. Five models were tested within a maximum‐likelihood framework using the LASER package. Two of these models –PB (pure birth) and BD (birth–death) – assume constant rates, while the remaining models allow for temporal rate variability: DDL (linear diversity dependent), DDX (exponential diversity dependent), and y2r (two‐parameter Yule). We then computed and compared AIC scores to assess model fit (Fig. S6). Our sampling included all extant species within the *Roucela* complex and, therefore, incomplete taxon sampling should not be an issue in these analyses.

## Results

### Phylogenetic analyses

All analyses recovered the *Roucela* complex as monophyletic with the exclusion of the Balkan endemic, *C. scutellata*. This taxon falls well outside the *Roucela* clade, a result not surprising when considering its distinct morphology and chromosome number. The broader corolla and larger overall size of this species led Carlström (1986) to question its placement within *Roucela*. Our analyses corroborate Mansion et al. (2012) and place *C*. *scutellata* with other annuals in the *Megalocalyx* clade.

Both concatenation and species tree analyses were performed in order to compare results from these different methods. Although results were very similar, we chose to present both trees as the phylogeny resulting from the concatenated dataset provides relationships within species (between populations), allowing us to make inferences regarding biogeographic history of taxa and potential gene flow between species. Results from these analyses are discussed below.

#### Plastid

Our plastid dataset recovered a strongly supported *Roucela* clade sister to a clade containing the North African taxa *Feeria angustifolia* and *Campanula edulis*, the Azorian endemic *Azorina vidalii*, and *Campanula mollis*, distributed in the western Mediterranean (see Fig. S2). This placement within the Campanuloideae is consistent with the previous studies (Cellinese et al. 2009; Haberle et al. 2009; Mansion et al. 2012; Crowl et al. 2014).

Within the *Roucela* complex, three clades can be distinguished, corresponding to an “eastern grade” and a “western clade” (Fig. S2). The eastern grade is composed of two clades found only east of the MAT. The earliest diverging clade includes two species, *Campanula pinatzii* (found on Karpathos and Kasos) and the very narrow endemic *C. kastellorizana* (restricted to the island of Kastellorizo). The second eastern clade contains *C. raveyi* (western Turkey), *Campanula rhodensis* (endemic to Rhodes), *C. lycica* (western Turkey and Kastellorizo), *Campanula delicatula* (southwest Turkey, southeast Aegean, and Cyprus), *C. veneris* (narrowly endemic in the Troodos Mountains of Cyprus), and *C. podocarpa* (southwest Turkey, eastern Aegean Islands, and Cyprus).

The “western clade” includes two species found exclusively west of the MAT, *C. creutzburgii* (endemic to Crete) and *C. drabifolia* (mainland Greece), as well as the widespread *C. erinus* and one eastern species, *Campanula simulans* (eastern Aegean Islands and western Turkey).

*Campanula erinus* was inferred to be polyphyletic (Fig. S2). Interestingly, plastid markers found individuals of this taxon to fall into three clades, roughly corresponding to geographic regions. Support for relationships among *C. erinus* populations and other taxa in this clade, however, are not sufficient to draw meaningful conclusions.

#### Nuclear

Nuclear and plastid loci gave largely similar results (see Fig. S2). The placement of the *Roucela* complex within the Campanuloideae is consistent with both datasets. The nuclear dataset also recovered three clades within a monophyletic *Roucela*. Although the relationships among these clades are poorly supported, the content of the clades is largely consistent.

Paralogy was previously tested for the nuclear genes within this clade (Crowl et al. 2014), and therefore, incongruences in this study are likely the result of incomplete lineage sorting and/or hybridization. We recovered one likely instance of hybridization. Two accessions of *C. lycica* are found to be sister to *C. kastellorizana* in the nuclear phylogeny. These taxa are sympatric on the island of Kastellorizo, where these individuals were collected, suggesting the inconsistent placement of *C. lycica* between nuclear and plastid markers may be the result of interspecific gene flow.

#### Species tree and combined dataset

Results from the independent plastid and nuclear loci did not show significant (highly supported) incongruences; therefore, we combined all loci into a single dataset. The concatenated dataset generated similar results to the plastid‐only analyses, but with increased support for many relationships. As an alternative, results from the concatenated dataset were compared to a species tree. Our concatenated and species tree analyses recovered equivalent species relationships (with one exception) within a strongly supported *Roucela* clade (Fig. 2).

Similar to the plastid‐only results, we found three well‐supported clades that correspond to an eastern grade and a western clade. Relationships within these clades are nearly identical to those obtained with the plastid‐only dataset. By concatenating plastid and nuclear loci, however, we were able to increase resolution toward the terminals of the phylogeny, uncovering intraspecific (population‐level) structure.

This dataset recovered two clades within *C. podocarpa*: One contains only accessions from the Turkish mainland, while the other includes only individuals from Cyprus. A sister relationship is inferred for *C. podocarpa* and the narrow Cyprus endemic, *C. veneris*. Similarly, within the *C. delicatula* clade, we recovered a grade of Turkish accessions sister to a clade (although poorly supported) of Aegean populations.

Resolution within the western clade was not significantly improved in the combined dataset. Although we recovered increased support for the monophyly of *C. simulans*, this dataset did not provide definitive results regarding relationships within the clade or monophyly of *C. creutzburgii*,* C. drabifolia*, or *C. erinus*. The species tree analysis, however, recovered well‐supported relationships for all four taxa. This analysis found the widespread *C. erinus* to be sister to *C. creutzburgii*, a species endemic to the island of Crete. However, all other analyses (non–species‐tree methods) recovered *C. erinus* as polyphyletic with respect to *C. creutzburgii* or *C. drabifolia*.

### Divergence time estimates and ancestral range estimation

Divergence estimates obtained from a concatenated dataset of the broader Campanuloideae clade generated ages older than expected given the low level of morphological divergence between *Roucela* taxa. This is congruent with the results of Mansion et al. (2012). The *Roucela* clade is inferred to have originated in the early Miocene (Fig. 3). The early diverging eastern clade dates to approximately 22 Ma. The split between the western and eastern clades occurred approximately 19 Ma.

Ancestral range estimation using BioGeoBEARS recovered the DEC model as the best‐fit model, and inclusion of the founder‐event parameter (+J) significantly (*P* < 0.05) improved the log likelihood of this model for all analyses. The time‐stratified analysis excluding *C. erinus* was inferred to be significantly better than other analyses. Summary statistics for all models tested are provided in Fig. S4.

The origin of the *Roucela* clade was inferred as Karpathos + Rodos + Turkey + the eastern Aegean (Fig. 3). During the early Miocene, all of these areas were connected as a single landmass, suggesting the eastern Aegean landmass as the ancestral range. Multiple vicariance and dispersal events were inferred during cycles of island connection and isolation as this landmass fragmented (Fig. 3).

### Niche modeling

Of the climatic layers included in this study, elevation, mean temperature of coldest quarter, precipitation of wettest quarter, precipitation of driest quarter, mean diurnal range, temperature seasonality, minimum temperature of coldest month, and mean temperature of wettest quarter were found to be least correlated.

Using the above climatic layers and current distribution data, niche modeling results recovered an average AUC score of 0.926–0.950, with standard deviations from 0.030 to 0.065, suggesting low rates of inaccurate predictions.

**Figure 4:**
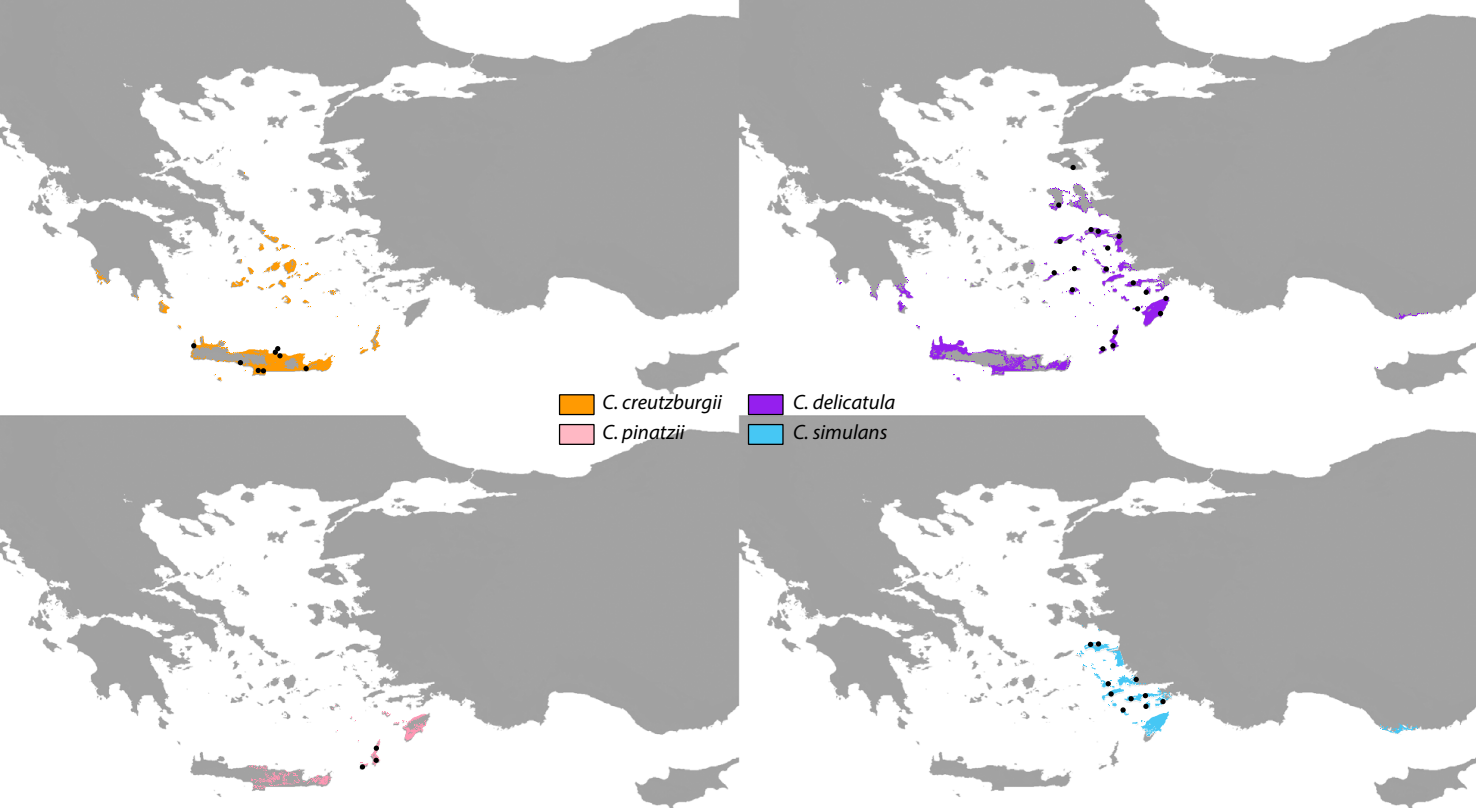
Niche modeling. Results from climatic niche modeling analyses for four *Roucela* taxa: (A) *Campanula creutzburgii*, (B) *Campanula delicatula*, (C) *Campanula pinatzii*, and (D) *Campanula simulans*. Colors represent inferred fundamental climatic niche space for each species. Black dots indicate representative occurrence points to indicate approximate, current distributions. Results suggest realized distributions represent a subset of fundamental climatic niche space for all taxa tested.

Geographically, fundamental niches appear to be broader than realized niches in all species tested (Fig. 4). In other words, realized niches appear to be a subset of the fundamental niche space and there exists unoccupied, potentially suitable habitat beyond the current distribution of all taxa.

### Diversification

**Figure 5:**
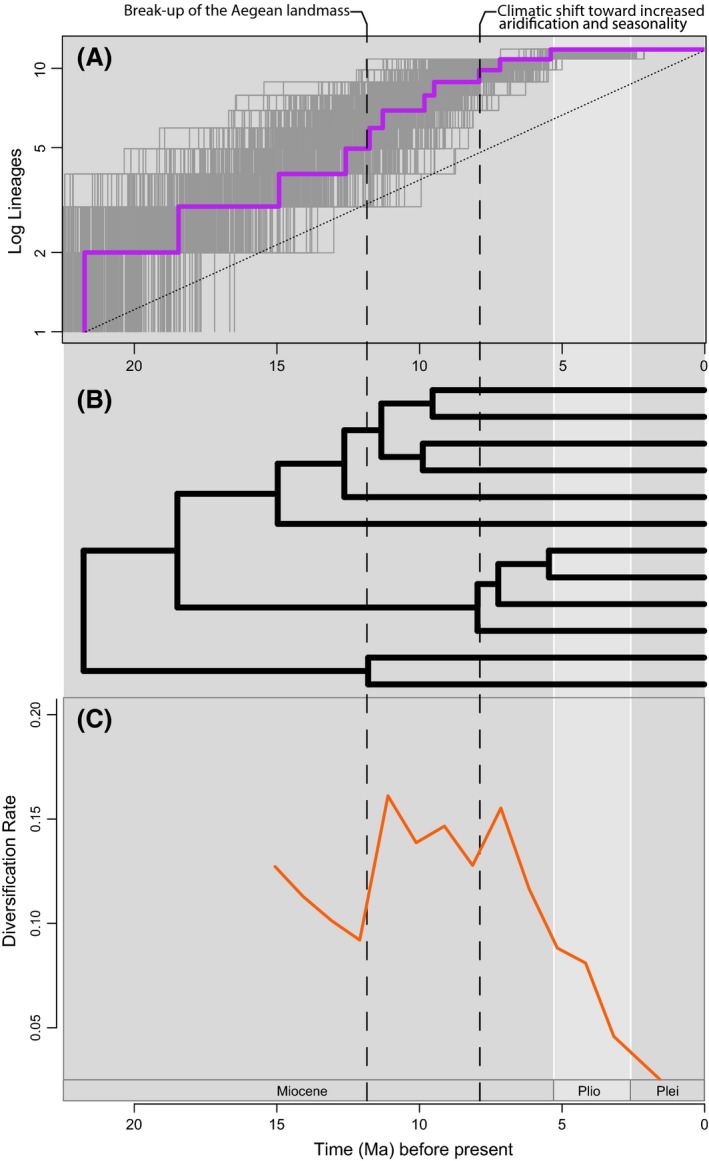
Tempo and pattern of diversification of the *Roucela* clade. Results from diversification analyses. (A) Log lineage‐through‐time (LTT) plot. LTT plots for the posterior distribution of trees from BEAST analysis (post‐burn‐in) shown in gray. Dotted line indicates hypothetical constant diversification. (B) Chronogram for the *Roucela* clade from BEAST analysis. *Campanula erinus* reduced to a single accession to approximate the species tree topology. (C) Diversification rate through time using the sliding window approach of Simpson et al. (2011). Diversification rate is calculated as the number of nodes over the sum of all branch lengths within a window of given length.

The LTT plot shows a rapid accumulation of lineages through the mid‐Miocene and then quickly tapers off beginning approximately 8–7 Ma (Fig. 5). We obtained a gamma value of −2.2976 and a *P*‐value of 0.01, indicating we can reject the null hypothesis of “rates have not decreased over time” for this clade. The large negative value for the gamma statistic can be interpreted as evidence for a decrease in speciation rate through time. The LTT plot and sliding window analysis verify this. Results from the sliding window method indicate a burst of speciation between approximately 12 Ma and 8 Ma (Fig. 5). This is followed by a decline in diversification, persisting to the present.

We obtained AIC values for the diversification models listed above ranging between 33.864 and 43.073 (Fig. S6). Our results, based on the AIC score for each model, suggest that the DDL (logistic diversity dependent; AIC = 33.864) model and the y2r (two‐rate Yule; AIC = 35.605; st = 8.9) are the best fit to the data. Because these models are within 2 AIC values of each other, we consider both equally likely (Burnham and Anderson 2002).

## Discussion

### Evolution and biogeography of the *Roucela* clade

Both dispersal and vicariance appear to be historically important processes in driving the biogeographic patterns we observe in the *Roucela* clade. The group likely originated from a Eurasian ancestor (see also Mansion et al. 2012) during the early Miocene when the area was experiencing a subtropical climate (Fig. 3). Contrary to the past studies of Mediterranean biota (e.g., Yesson and Culham 2006; Mijarra et al. 2009; Fiz‐Palacios and Valcárcel 2013), our results indicate the onset of the Mediterranean climate has not promoted diversification within the clade. In fact, the shift to increased seasonality and decreased rainfall appears to have greatly slowed the rate of diversification (Fig. 5). Speciation was, instead, likely the result of ancient geologic and tectonic events, which led to numerous cycles of island connection and isolation (Fig. 3).

Dating and diversification analyses indicate the timing of diversification within the *Roucela* clade coincides with the breakup of an ancient landmass (Ägäis) in the Miocene, suggesting vicariance as a likely driver of diversification in the clade (Figs. 3, 5). Additionally, we found geologic events such as the formation of the MAT and the MSC to be historically important in the evolutionary history of this group.

#### Breakup of Aegean landmass

The complex geologic history of the Aegean has played a significant role in shaping biogeographic patterns in the area. One such event is the fragmentation of an ancient landmass during the middle Miocene. Tectonic movements and changing sea levels, beginning approximately 12 Ma, caused the Aegean landmass to break apart – eventually forming the continental island system of the Aegean Archipelago (Dermitzakis and Papanikolaou 1981).

Molecular dating and ancestral range estimation suggest the *Roucela* clade originated while this landmass was still intact, and we find evidence that much of the diversification within the group is the result of vicariance caused by its breakup (Figs. 3, 5). Specifically, *C. drabifolia*,* C. rhodensis*,* C. pinatzii*, and *C. kastellorizana* were found to be the result of vicariance driven by rising sea levels and continental fragmentation (Fig. 3).

The separation of the eastern and western Aegean, approximately 12–9 Ma, led to the formation of the MAT (Creutzburg 1963; Dermitzakis 1990), which has long acted as a barrier to dispersal and has been hypothesized to be a major factor in the evolutionary history of many taxa, including reptiles (Poulakakis et al. 2005, 2008, 2013; Lymberakis and Poulakakis 2010), scorpions (Parmakelis et al. 2006), land snails (Kornilios et al. 2009), and plants (Strid 1996; Bittkau and Comes 2005). This formation appears to be important in the current species distribution within the *Roucela* clade.

*Roucela* taxa show a clear geographic pattern and can be divided into eastern and western species relative to the MAT, with the exception of *C. erinus*, which is widespread throughout the Mediterranean and *C*. *delicatula*, which occurs primarily east of the trench but is also distributed on islands just west of it (Carlström 1986).

Dating analysis indicates that *C*. *delicatula* diverged prior to the formation of the MAT (Fig. 3), giving a possible explanation for the distribution of this taxon. However, this species seems to have dispersed to Cyprus (see below), suggesting that dispersal over this barrier cannot be discounted as a possibility. Unfortunately, individuals from west of the trench were not available to test these hypotheses.

All analyses confirm the presence of three distinct clades within the group. Two clades composed exclusively of eastern taxa form a grade sister to a clade containing two western species (*C. creutzburgii* and *C*. *drabifolia*), the widespread *C. erinus*, and one eastern species, *C*. *simulans* – endemic to western Turkey and adjacent islands.

The placement of *C*. *simulans* within the western clade is peculiar as the divergence of this species occurred after the formation of the MAT, suggesting it is the result of a single dispersal event to the eastern Aegean, across this barrier (Figs. 2, 3). However, our results suggest this dispersal occurred during the MSC, when many landmasses in the eastern Aegean were again connected due to a drastic sea‐level drop (Fig. 3). The MAT may have represented a less significant barrier to dispersal during this time.

#### Cyprus disjunctions

Cyprus has a complex paleogeographic history. Past researchers (e.g., Schmidt 1960) considered it a continental island, a landmass once connected to the mainland. However, more recent studies have shown this island to be of oceanic origins, formed at the junction of the Eurasian and African plates (Gass 1968; Moores and Vine 1971) and one of the most isolated islands (both geologically and biologically) in the Mediterranean Basin (Moores et al. 1984).

In addition to the widespread *C. erinus,* three *Roucela* taxa are found on Cyprus: *C. veneris*,* C. delicatula*, and *C. podocarpa* (Fig. 1). *Campanula delicatula* and *C. podocarpa* show narrow, disjunct distributions between western Turkey and Cyprus. Interestingly, these taxa are sympatric in western Turkey but have nonoverlapping distributions on Cyprus. *Campanula veneris* is a narrow Cypriot endemic, found only in the Troodos Mountains, where it is parapatric with *C. podocarpa*.

The current, nonoverlapping distributions of *C. delicatula* and *C. podocarpa* on Cyprus are likely the result of edaphic differences and the separate geologic histories of the two landmasses comprising the present day island. The apparent low‐dispersal ability of these taxa suggests geodispersal events during a time of low sea level.

Results from dating analyses estimate the split between Turkish accessions of *C. podocarpa* and individuals from Cyprus at approximately 5 Ma (Fig. S3), suggesting dispersal (or range expansion) of this taxon during the MSC, when “stepping‐stone” islands may have connected the two islands to the mainland (Hadjisterkotis et al. 2000). Divergence of the Cypriot population of *C. delicatula* from the mainland accessions was estimated to occur less than one million years ago, suggesting dispersal of this taxon occurred during Pleistocene glacial cycles.

#### *Campanula erinus*

The evolutionary history of *C. erinus* remains enigmatic, and phylogenetic relationships continue to be largely unresolved. Species tree analyses indicate *C. erinus* is sister to the Cretan endemic, *C. creutzburgii*, while analyses of concatenated and individual gene datasets suggest it is polyphyletic and a close relationship with both *C. creutzburgii* and *C. drabifolia* is inferred (Fig. 2). Dating analyses indicate relatively young divergence dates for all the *C. erinus* individuals included in this study (with the exception of one Spanish accession), suggesting that the nonmonophyly may be the result of incomplete lineage sorting. However, phylogenetic placement of populations using plastid markers appears to follow a loose geographic pattern – indicating that it is possible this taxon represents a cryptic species complex containing multiple lineages of taxa exhibiting low levels of morphological divergence. Two chromosome numbers have been reported for *C. erinus* (2*n* = 28 and 56; Carlström 1986)), suggesting polyploid populations exist.

Whether the putative polyploids are the result of hybridization or autopolyploidy, the result of a single or multiple events, and whether *C. erinus* represents multiple, independent evolutionary lineages that have simply not been recognized by taxonomists is currently being investigated (A. Crowl and N. Cellinese, in prep.). Disentangling the historical processes that have led to the apparent nonmonophyly of *C. erinus* in this study promises to provide even further insights into the evolutionary processes in the Mediterranean Basin.

#### Niche modeling

Our results indicate that, for all taxa tested, the realized distributions of *Roucela* species are much narrower than potential niche space (Fig. 4). The narrow endemism of these taxa in the Eastern Mediterranean therefore does not appear to be the result of climatic constraints.

No discernible adaptations to dispersal have been observed in *Roucela* species. Field observations suggest seed dispersal by gravity is likely. This dispersal mechanism may also be partly responsible for the narrow distribution of these taxa. In other words, these endemic species have no way to effectively fill their fundamental niche.

#### Diversification

We reject the hypothesis of a constant rate of species diversification through time for the *Roucela* complex. The sliding window analysis indicates an elevated diversification rate between approximately 8 and 12 Ma (Fig. 5). This corresponds to the breakup of the Aegean landmass, further supporting our hypothesis that allopatric speciation caused by this event is likely responsible for much of the diversity in the *Roucela* clade. The diversification models found to be most significant for this dataset – the DDL and y2r model – provide two potential explanations regarding the apparent slowdown in diversification rate that follows.

The DDL model predicts speciation rates to be density dependent and, therefore, decline (linearly) through time as the number of lineages increase, and thus, ecological niche space becomes saturated (Rabosky and Lovette 2008).

The y2r model, on the other hand, suggests that the clade has diversified under a single rate until, at some point in time, a switch occurs and the clade begins diversifying at a new rate. The timing of this rate shift was estimated at approximately 8 Ma for the *Roucela* clade (Figs. 5, S6), corresponding to a trend toward increased aridification and away from a subtropical climate in the Mediterranean Basin, providing a likely explanation for decreased speciation in a clade adapted to subtropical conditions. It is important to note, however, that recent extinction cannot be discounted as a possible cause for the pattern we observe.

## Concluding Remarks

Our results provide detailed insights into the evolutionary history of *Campanula* species in the eastern Mediterranean. This area has had a complex, but well‐understood, geologic and climatic history. By reconstructing evolutionary relationships and estimating divergence times, we are able to test the relative importance of specific historical events that have contributed to the evolutionary history of the *Roucela* clade. We have shown that the evolutionary history and current distributional patterns of these taxa are the result of both dispersal and vicariance events and have been shaped by numerous events through the Miocene and onward.

Specifically, our results indicate the breakup of an ancient Aegean landmass to be responsible for driving diversification of these species. Contrastingly, climatic shifts beginning approximately 8 Ma, and eventually leading to the current Mediterranean climate, appear to be responsible for a decrease in diversification rate in the *Roucela* clade. Continental island endemics likely originated via vicariance, whereas the oceanic island endemic on Cyprus appears to be the product of a single dispersal event from the mainland, followed by *in situ* diversification. The narrow endemism of *Roucela* taxa does not appear to be due to climatic constraints, but is likely linked to the apparent low‐dispersal ability of this group.

By studying the *Roucela* complex in an evolutionary and biogeographic context, we highlight the diversity and complexity of historical processes driving plant evolution in the Mediterranean Basin.

## Conflict of Interest

None declared.

## Supporting information


**Figure S1.** Sample accessions.

**Figure S2.** Plastid, Nuclear, and Combined Trees.

**Figure S3.** Beast Chronogram for Campanuloideae.

**Figure S4.** Summary statistics for BioGeoBEARS models from three separate analyses.

**Figure S5.** Chronogram inferred using a multispecies coalescent approach (*BEAST).

**Figure S6.** Results from diversification analyses.
