# Supplementary material for: Evolution and biogeography of the endemic Roucela complex (Campanulaceae: Campanula) in the Eastern Mediterranean
Source: Ecol Evol. 2015 Oct 28;5(22):5329–43. doi: 10.1002/ece3.1791 (PMC6102515; doi:10.1002/ece3.1791)
Supplement: Supplementary file 2 — Figure S2. Plastid, Nuclear, and Combined Trees. [file ECE3-5-5329-s002.pdf]

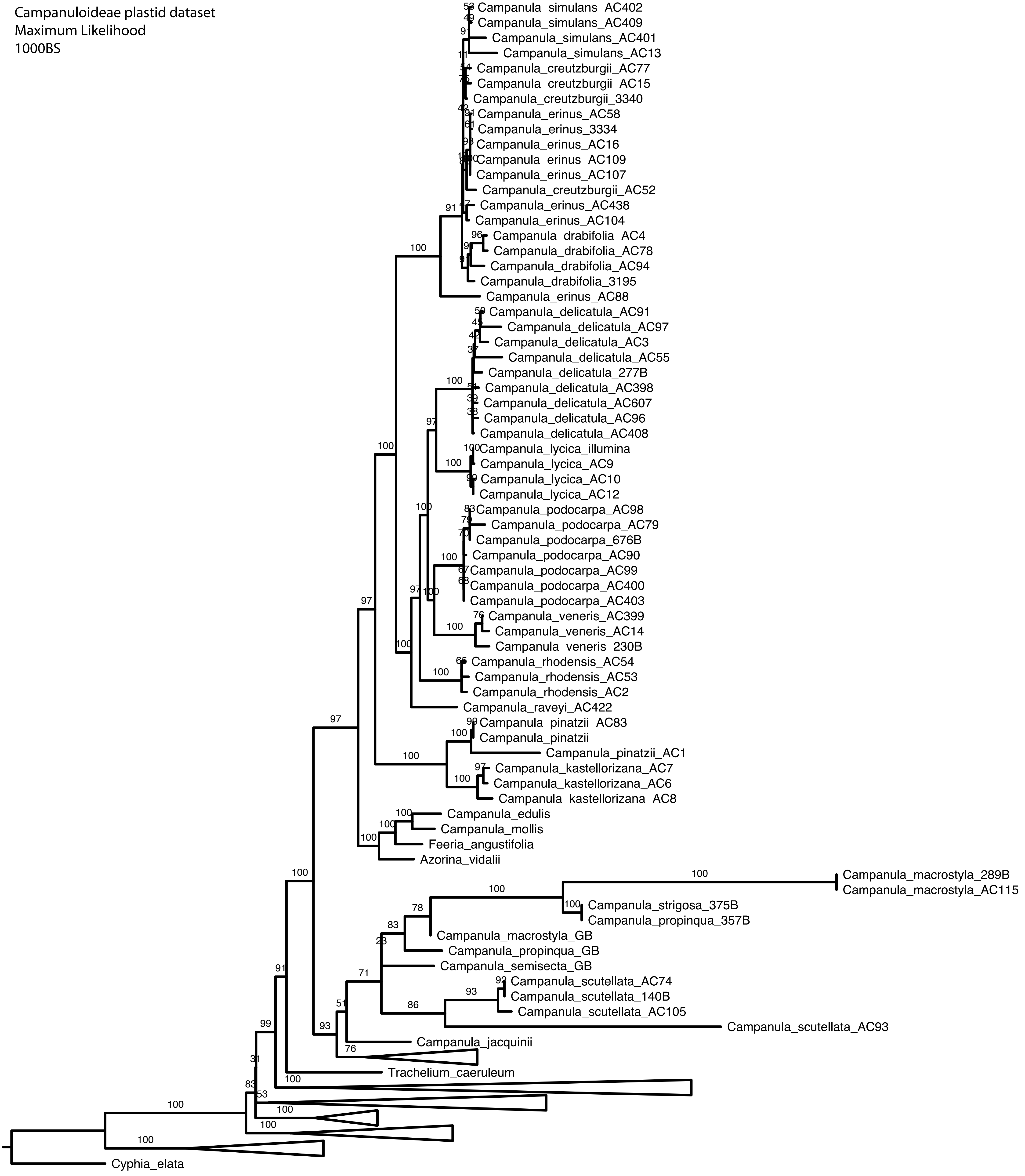
Campanuloideae plastid dataset
Maximum Likelihood
1000BS
Campanula_simulans_AC402
Campanula_simulans_AC409
Campanula_simulans_AC401
Campanula_simulans_AC13
Campanula_creutzburgii_AC77
Campanula_creutzburgii_AC15
Campanula_creutzburgii_3340
Campanula_erinus_AC58
Campanula_erinus_3334
Campanula_erinus_AC16
Campanula_erinus_AC109
Campanula_erinus_AC107
Campanula_creutzburgii_AC52
Campanula_erinus_AC438
Campanula_erinus_AC104
Campanula_drabifolia_AC4
Campanula_drabifolia_AC78
Campanula_drabifolia_AC94
Campanula_drabifolia_3195
Campanula_erinus_AC88
Campanula_delicatula_AC91
Campanula_delicatula_AC97
Campanula_delicatula_AC3
Campanula_delicatula_AC55
Campanula_delicatula_277B
Campanula_delicatula_AC398
Campanula_delicatula_AC607
Campanula_delicatula_AC96
Campanula_delicatula_AC408
Campanula_lycica_illumina
Campanula_lycica_AC9
Campanula_lycica_AC10
Campanula_lycica_AC12
Campanula_podocarpa_AC98
Campanula_podocarpa_AC79
Campanula_podocarpa_676B
Campanula_podocarpa_AC90
Campanula_podocarpa_AC99
Campanula_podocarpa_AC400
Campanula_podocarpa_AC403
Campanula_veneris_AC399
Campanula_veneris_AC14
Campanula_veneris_230B
Campanula_rhodensis_AC54
Campanula_rhodensis_AC53
Campanula_rhodensis_AC2
Campanula_raveyi_AC422
Campanula_pinatzii_AC83
Campanula_pinatzii
Campanula_pinatzii_AC1
Campanula_kastellorizana_AC7
Campanula_kastellorizana_AC6
Campanula_kastellorizana_AC8
Campanula_edulis
Campanula_mollis
Feeria_angustifolia
Azorina_vidalii
Campanula_macrostyla_289B
Campanula_macrostyla_AC115
Campanula_strigosa_375B
Campanula_propinqua_357B
Campanula_macrostyla_GB
Campanula_propinqua_GB
Campanula_semisecta_GB
Campanula_scutellata_AC74
Campanula_scutellata_140B
Campanula_scutellata_AC105
Campanula_scutellata_AC93
Campanula_jacquinii
Trachelium_caeruleum
Cyphia_elata

Campanuloideae nuclear dataset
Maximum Likelihood
1000BS

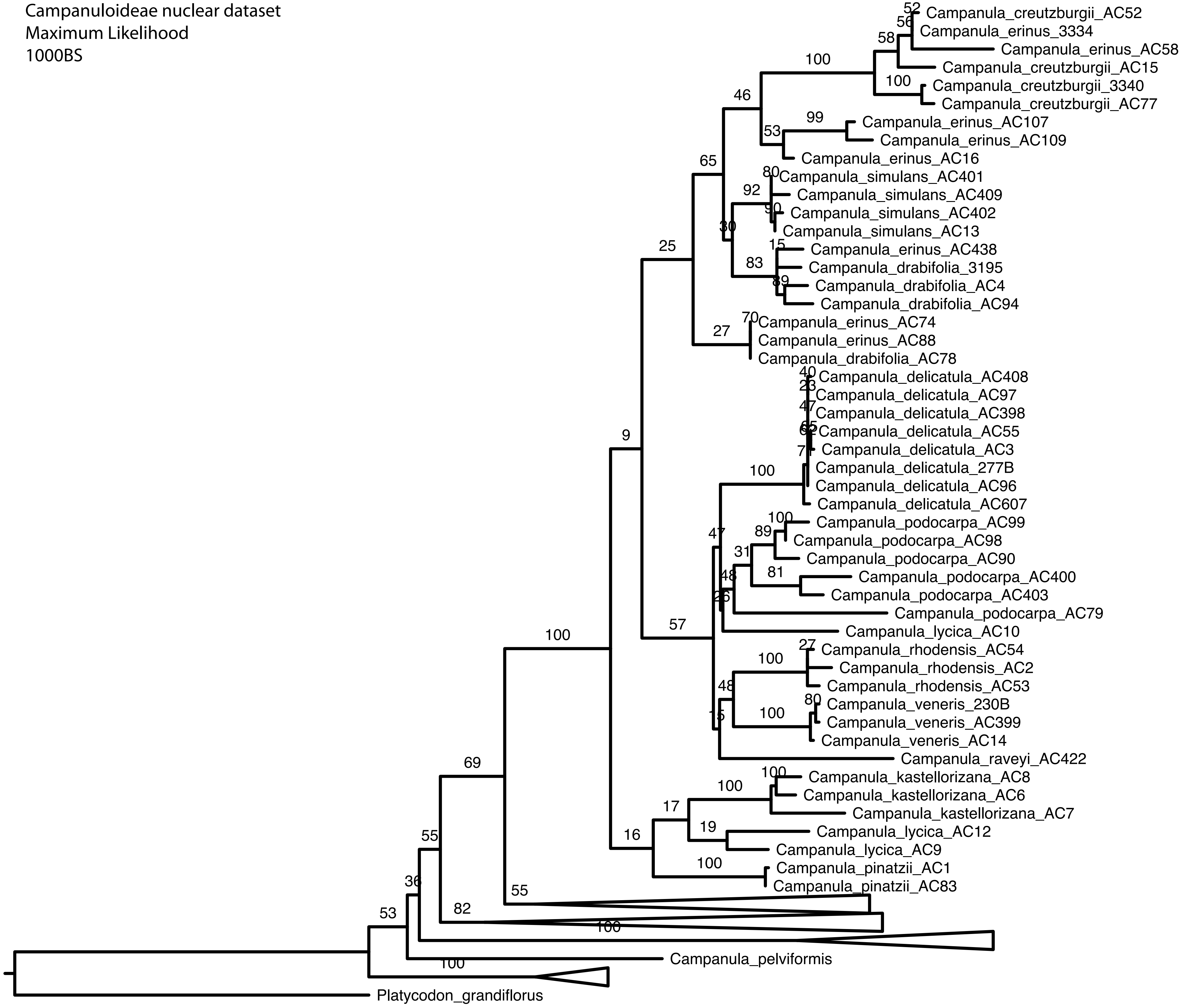

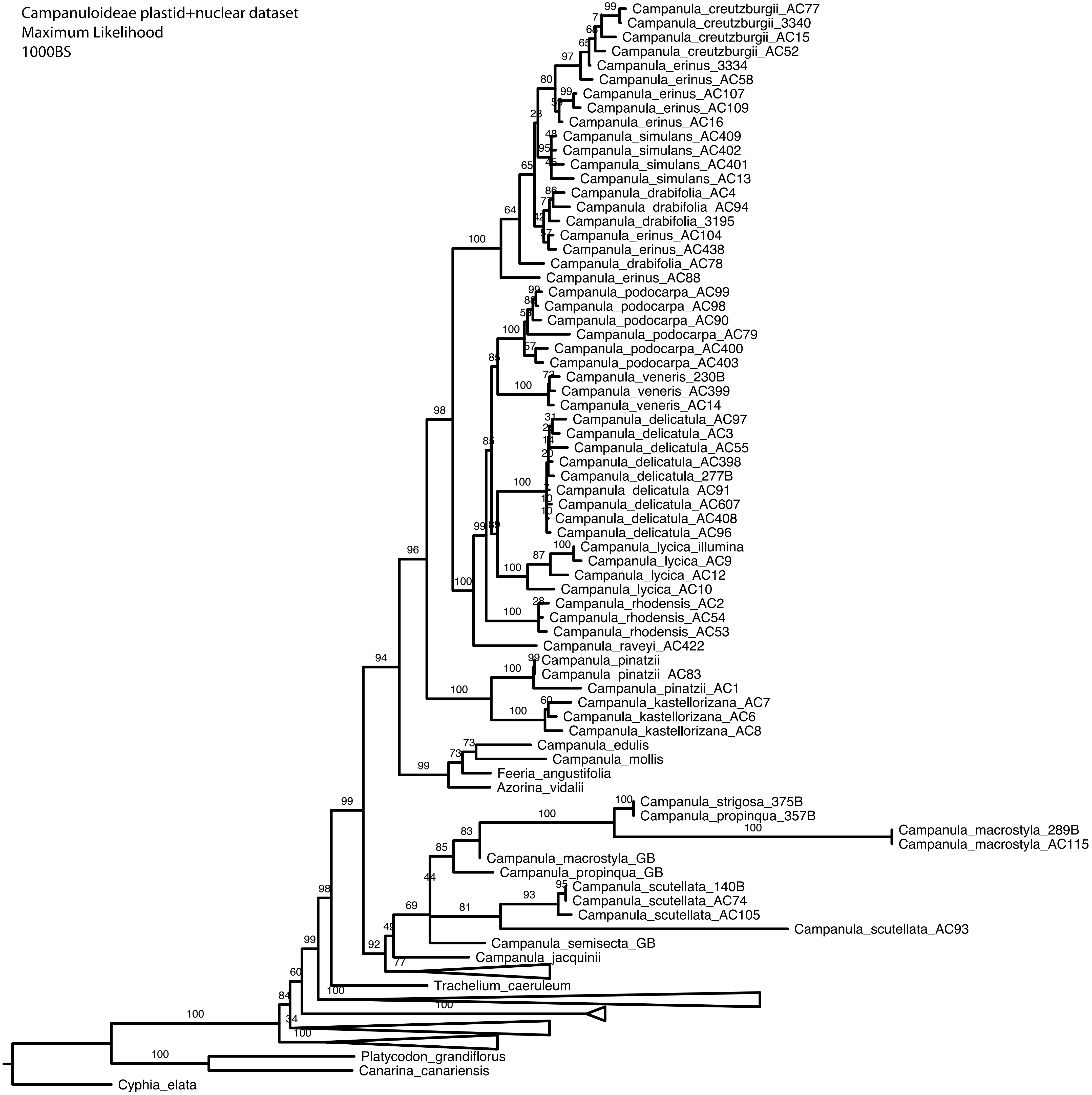
Campanuloideae plastid+nuclear dataset
Maximum Likelihood
1000BS
Campanula_creutzburgii_AC77
Campanula_creutzburgii_3340
Campanula_creutzburgii_AC15
Campanula_creutzburgii_AC52
Campanula_erinus_3334
Campanula_erinus_AC58
Campanula_erinus_AC107
Campanula_erinus_AC109
Campanula_erinus_AC16
Campanula_simulans_AC409
Campanula_simulans_AC402
Campanula_simulans_AC401
Campanula_simulans_AC13
Campanula_drabifolia_AC4
Campanula_drabifolia_AC94
Campanula_drabifolia_3195
Campanula_erinus_AC104
Campanula_erinus_AC438
Campanula_drabifolia_AC78
Campanula_erinus_AC88
Campanula_podocarpa_AC99
Campanula_podocarpa_AC98
Campanula_podocarpa_AC90
Campanula_podocarpa_AC79
Campanula_podocarpa_AC400
Campanula_podocarpa_AC403
Campanula_veneris_230B
Campanula_veneris_AC399
Campanula_veneris_AC14
Campanula_delicatula_AC97
Campanula_delicatula_AC3
Campanula_delicatula_AC55
Campanula_delicatula_AC398
Campanula_delicatula_277B
Campanula_delicatula_AC91
Campanula_delicatula_AC607
Campanula_delicatula_AC408
Campanula_delicatula_AC96
Campanula_lycica_illumina
Campanula_lycica_AC9
Campanula_lycica_AC12
Campanula_lycica_AC10
Campanula_rhodensis_AC2
Campanula_rhodensis_AC54
Campanula_rhodensis_AC53
Campanula_raveyi_AC422
Campanula_pinatzii
Campanula_pinatzii_AC83
Campanula_pinatzii_AC1
Campanula_kastellorizana_AC7
Campanula_kastellorizana_AC6
Campanula_kastellorizana_AC8
Campanula_edulis
Campanula_mollis
Feeria_angustifolia
Azorina_vidalii
Campanula_strigosa_375B
Campanula_propinqua_357B
Campanula_macrostyla_289B
Campanula_macrostyla_AC115
Campanula_macrostyla_GB
Campanula_propinqua_GB
Campanula_scutellata_140B
Campanula_scutellata_AC74
Campanula_scutellata_AC105
Campanula_scutellata_AC93
Campanula_semisecta_GB
Campanula_jacquinii
Trachelium_caeruleum
Platycodon_grandiflorus
Canarina_canariensis
Cyphia_elata
