# Supplementary figures and images for: Evolution and biogeography of the endemic Roucela complex (Campanulaceae: Campanula) in the Eastern Mediterranean
Source: Ecol Evol. 2015 Oct 28;5(22):5329–43. doi: 10.1002/ece3.1791 (PMC6102515; doi:10.1002/ece3.1791)

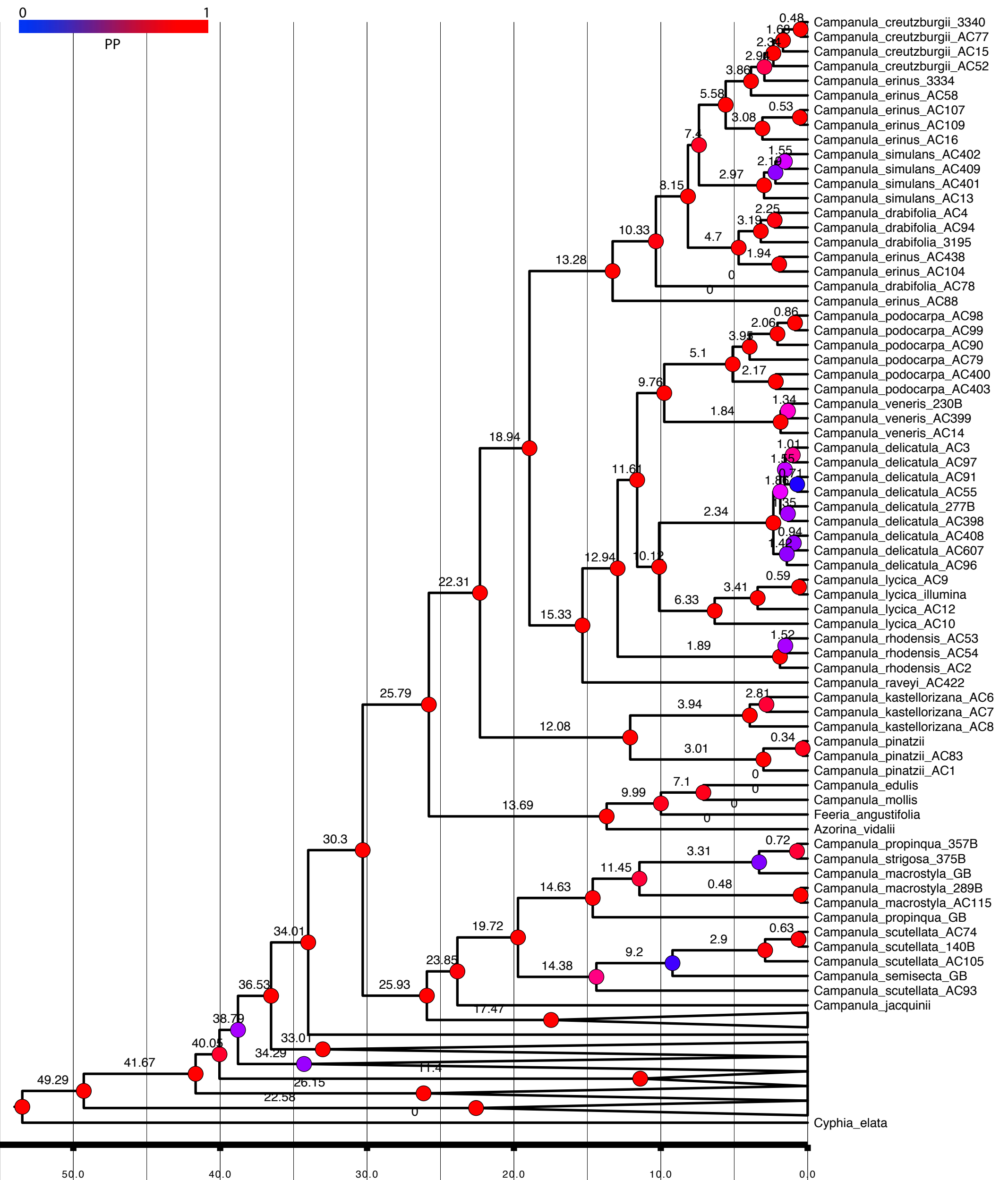

Supplement: Supplementary file 3 — Figure S3. Beast Chronogram for Campanuloideae. [file ECE3-5-5329-s003.pdf]
