# Supplementary material for: Evolution and biogeography of the endemic Roucela complex (Campanulaceae: Campanula) in the Eastern Mediterranean
Source: Ecol Evol. 2015 Oct 28;5(22):5329–43. doi: 10.1002/ece3.1791 (PMC6102515; doi:10.1002/ece3.1791)

Fig. S5 Chronogram inferred using a coalescent species tree approach in *BEAST. Results from this analysis provided divergence dates an order of magnitude younger than other methods and previous studies of the group. Numbers above branches are divergence dates in millions of years ago.

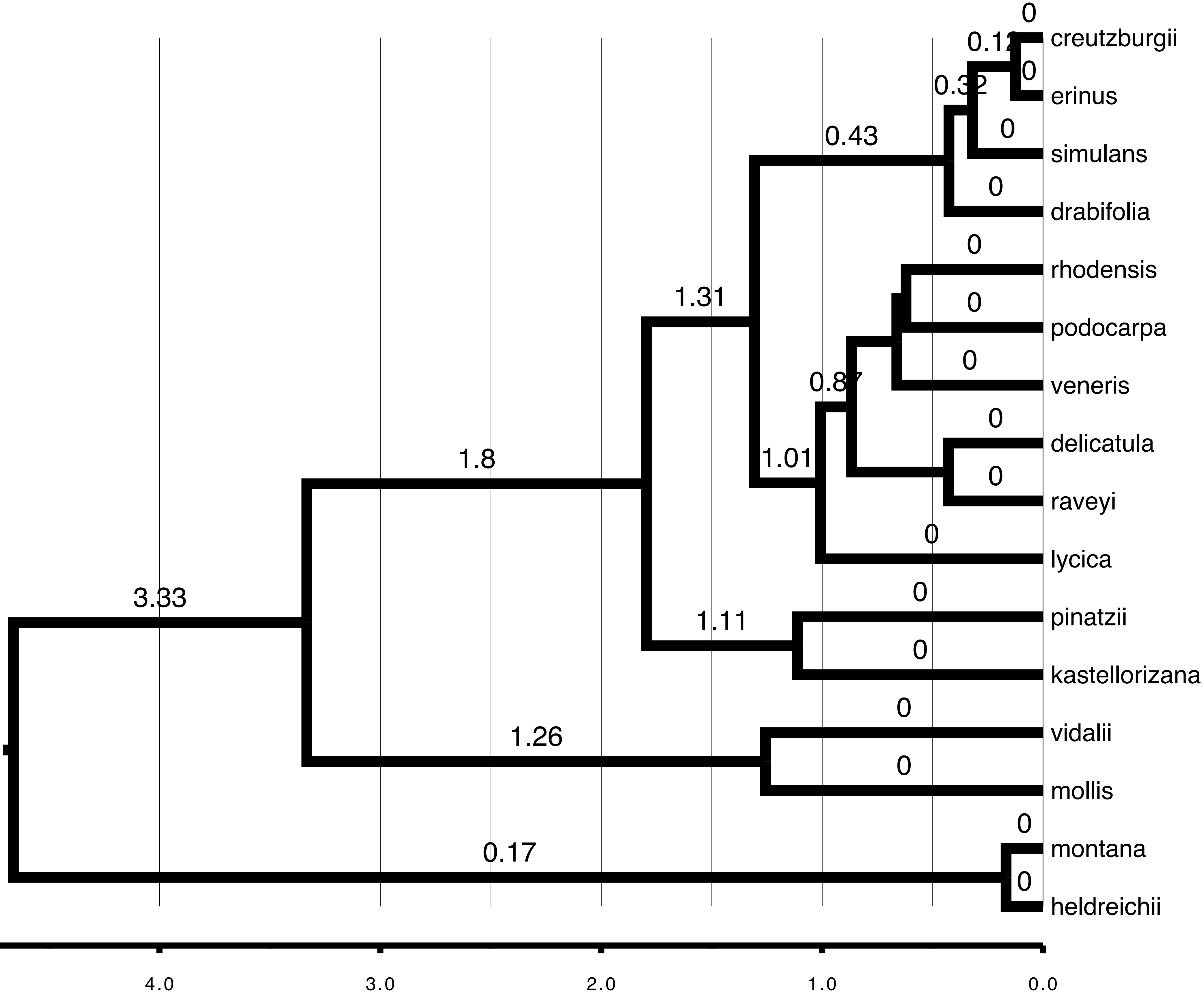

Supplement: Supplementary file 5 — Figure S5. Chronogram inferred using a multispecies coalescent approach (*BEAST). [file ECE3-5-5329-s005.pdf]
