# Supplementary material for: Evolution and biogeography of the endemic Roucela complex (Campanulaceae: Campanula) in the Eastern Mediterranean
Source: Ecol Evol. 2015 Oct 28;5(22):5329–43. doi: 10.1002/ece3.1791 (PMC6102515; doi:10.1002/ece3.1791)
Supplement: Supplementary file 6 — Figure S6. Results from diversification analyses. [file ECE3-5-5329-s006.docx]

Figure S6. Results from the diversification analyses of the *Roucela* clade. Models were fit to the chronogram generated by BEAST. AIC = Aikaike Information Criterion; r = net diversification rates (speciation events / million years); k = carrying capacity; x = rate change parameter; st = estimated time of rate shift (in million of years before present).

| Diversification model | Model type | LH | AIC | Parameter estimates |
| --- | --- | --- | --- | --- |
| Pure-birth | rate const. | -19.53659 | 41.07318 | r1=0.0669 |
| Birth-death | rate const. | -19.53659 | 43.07318 | r1=0.0669 |
| DDL | rates variable | -14.932 | 33.86401 | r1=0.3166 k=11.5367 |
| DDX | rates variable | -17.72157 | 39.44314 | r1=0.4273 x=0.9554 |
| yule2rate | rates variable | -14.80251 | 35.60502 | r1=0.1639 r2=0.0198 st=8.987 |
