# Supplementary material for: Evolution and biogeography of the endemic Roucela complex (Campanulaceae: Campanula) in the Eastern Mediterranean
Source: Ecol Evol. 2015 Oct 28;5(22):5329–43. doi: 10.1002/ece3.1791 (PMC6102515; doi:10.1002/ece3.1791)
Supplement: Supplementary file 7 [file ECE3-5-5329-s007.docx]

**Figure S1.** Sample accessions.

**Figure S2.** Plastid, Nuclear, and Combined Trees.

**Figure S3.** Beast Chronogram for Campanuloideae.

**Figure S4.** Summary statistics for BioGeoBEARS models from three separate analyses.

**Figure S5.** Chronogram inferred using a multispecies coalescent approach (*BEAST).

**Figure S6.** Results from diversification analyses.
